# Parenting Styles, Depressive Symptoms, and Problematic Online Game Use in Adolescents: A Developmental Cascades Model

**DOI:** 10.3389/fpubh.2021.710667

**Published:** 2021-09-21

**Authors:** Xiong Gan, Hao Li, Mengmeng Li, Chengfu Yu, Xin Jin, Congshu Zhu, Yifan Liu

**Affiliations:** ^1^Department of Psychology, College of Education and Sports Sciences, Yangtze University, Jingzhou, China; ^2^Department of Psychology and Research Center of Adolescent Psychology and Behavior, School of Education, Guangzhou University, Guangzhou, China; ^3^Department of Psychology, College of Education and Sports Sciences, Yangtze University College of Technology and Engineering, Jingzhou, China

**Keywords:** parenting style, depressive symptoms, problematic online game use, developmental cascades, adolescence

## Abstract

Abundant empirical research has demonstrated the relationship between parenting style and adolescent problematic online game use (POGU), but the direction and underlying mechanism of this association remain unclear. Using a 1-year longitudinal design across three time points, the present study explored interrelations among parenting styles, depressive symptoms, and POGU from the theoretical perspective of the developmental cascade model and examined whether depressive symptoms mediate the relationship between parenting style and POGU. A sample of 1,041 children was recruited from two junior middle schools in China, of which 46.3% were boys. Results confirmed the cascade effects and showed that the reciprocal effect of parenting style, depressive symptoms, and POGU was significant, and parental control and POGU can predict each other *via* depressive symptoms. Knowledge regarding the direct and underlying mechanisms between parenting style, depressive symptoms, and POGU provides reference suggestions for the prevention and intervention of adolescent depressive symptoms and problematic online game use.

## Introduction

Since 1969, with the unique characteristics of high speed and low cost, the Internet has made rapid development and penetrated into all aspects of contemporary social life. As of December 2020, the number of Internet users in China has reached 989 million, while the number of online game users has reached 518 million, including more than 100 million young users (1). Problematic online game use (POGU) is a subtype of problematic Internet use and is defined as the “uncontrollable, excessive, and compulsive use of online games that causes serious problems such as social and/or emotional problems” (2). As POGU has become a global public health issue, it has been included in the updated version of the Diagnostic and Statistical Manual Disorders (DSM-5) and the International Classification of Diseases (ICD-11) and is thus attracting enormous attention from researchers (3, 4). An increasing number of empirical studies indicate that POGU affects the cognitive functioning of adolescents as well as their mental and physical health, contributing to negative outcomes, such as lack of self-confidence, academic failure, poor sleep quality, and violent crime (5, 6). In November 2019, the National Press and Publication Administration issued a notice on preventing minors from indulging in online games, focusing on “resolutely curbing addiction and protecting the healthy growth of teenagers” (7). This sparked widespread concern among people from all walks of life. The current important topic of discussion is based on how to guide teenagers to use the Internet in a proper manner, especially with respect to online games, to promote their healthy growth.

Developmental psychology pays attention to the social environment, family upbringing, parent–child relationship, and other environmental factors behind adolescent POGU behavior. As the most important social support system and resource of individuals, family plays a major role in the development of teenagers. According to the developmental system theory, behavior is a specific form of expression, and there is a set of interactive processes that leads to the current behavioral representation (8). Previous studies demonstrated that parenting style is not only a significant family factor affecting problematic online (game) use in teenagers (9) but also can affect the mental health of adolescents (e.g., depression) as part of the family ecological subsystem (10). Developmental contextualism indicates that the family ecological subsystem and individual subsystem influence each other (11), and similarly, children's behavior and mental health are also affected by parenting styles. Empirical studies showed that parenting style is closely related to problematic Internet use, which is not limited to POGU (12, 13). However, previous research has focused on the unidirectional influence of parenting style on adolescent POGU, and little is known about the bidirectional nature of this association and its underlying mechanism. Moreover, the mechanism of depressive symptoms between parenting style and adolescent POGU has not been investigated yet. Furthermore, this field mostly involves cross-sectional research, and there is a lack of discussion on the longitudinal relationship of variables and a clear explanation of the long-term predictive effects among variables. Hence, we intend to construct an autoregressive cross-lagged model to examine the direct and reciprocal association among parenting style, depressive symptoms, and adolescent POGU, as well as the potential mediating mechanisms among Chinese adolescents, to better understand these developmental processes and provide valuable information to help design effective prevention and intervention programs.

## Bidirectional Association Between Parenting Style and Adolescent POGU

Parenting style refers to the relatively stable attitude and behavior displayed by parents during the process of raising their children (14). According to the contextual–developmental perspective, the social behavior of teenagers is evaluated in the context of cultural norms and values (15). Parents attach importance to behaviors consistent with cultural norms and then influence the response to their children's character and behavior (16). In comparison with the West, Chinese culture emphasizes stringent adherence to group norms, commitment to family and social responsibilities, and behavioral and emotional constraints, which are more common among Chinese teenagers (17). Research shows that authoritarian parenting, overprotection, and rejection can positively predict adolescent POGU (9, 18). This is because teenagers are characterized by the search for autonomy and independence, and improper parenting style may interfere with their normal development of “separation–individuation,” and reduce parent-child relationship and self-esteem, so as to reduce autonomy in teenagers, thus leading to POGU (9). On the other hand, Patterson's social coercion theory (19) points out that adolescent problem behavior can also influence parenting style. Although no research has shown that adolescent POGU can predict parenting style, some studies have shown that adolescent externalized problem behavior can affect parenting style (20). Therefore, we speculate that parenting style and POGU can predict each other.

## Bidirectional Association Between Parenting Style and Depressive Symptoms

Negative parenting style can not only further aggravate problem behaviors in adolescents (e.g., substance abuse, cyberbullying, and POGU) (21–23) but also significantly increase the likelihood of internalizing problems (such as anxiety and depression) in adolescents (24). Cultural psychology maintains that human psychology and behavior are determined and restricted by culture, and emphasizes on the development and inheritance of human cultural history (25). Deeply influenced by Confucian culture, the Chinese praise introversion and forbearance and tend to suppress negative emotions in their hearts. According to the statistics released by the WHO, as of 2017, there are more than 350 million people in the world suffering from depression, of which more than 54 million people are in China (26). Adolescence is a critical period for the gradual enhancement of individual self-awareness and the continuous maturity of cognition, emotion, and personality, and hence mental health problems cannot be ignored (27). The National Commission and other 12 departments in China jointly formulated and issued “Healthy China Initiative—Action Plan for Mental Health of Children and Adolescents (2019–2022),” which emphasizes that schools and the concerned departments should actively organize mental health education activities to encourage parents to improve the mental health of their children by adopting a positive and correct parenting style (28). According to stress-buffer model, high levels of social support can help adolescents in relieving their stress, thereby reducing the likelihood of depressive symptoms (29). Many researchers have found that parental care and support negatively predict adolescent depressive symptoms (30–32). Additionally, adolescent depressive symptoms can also influence parenting style. Bronfenbrenner (33) believed that all the factors that affect parental behavior and children's development constitute a complete ecosystem, and these factors in turn affect each other within the ecosystem. Previous studies found that internalization of adolescents and parenting style can predict each other (30, 34). Therefore, we speculate that parenting style and depressive symptoms can predict each other.

## Bidirectional Association Between Depressive Symptoms and Adolescent POGU

The Internet has the ability to provide teenagers with social support, a sense of achievement, and the control they need, so that they can escape from the real world filled with emotional difficulties into the virtual world, and hence teenagers with depressive symptoms are more likely to use the Internet to relieve their stress, which in turn leads to POGU (35). On the other hand, studies have shown that adolescents with POGU often neglect interaction with their family and friends, resulting in more mental health problems (e.g., anxiety, depression, and loneliness) (36). In addition, a longitudinal study demonstrated that adolescent problematic video game use can predict depression and anxiety symptoms 2 years later (37). Therefore, we speculate that depressive symptoms and POGU can predict each other.

## Cascading Effects

Developmental cascades model proposes that the accumulation of many interactions leads to diffusion effects between different levels and different fields at the same level in the development of an individual (38). In other words, functioning in one domain influences functioning in other domains, resulting in the spreading of effects across domains over time. The developmental cascades model is a theoretical model constructed based on the cascade relationship between variables. Compared with the traditional longitudinal mediation model, the developmental cascades model considers correlation between variables concurrently and potential relations among variables longitudinally between adjacent time points.

Some longitudinal studies have shown that parenting style, depressive symptoms, and POGU can predict each other over time. For example, the parenting style at an earlier time can predict an internalizing problem (e.g., depression) occurring after 1 year (39), and that internalizing problem (e.g., depression) can significantly predict the subsequent parenting attitude and behavior (34), which in turn lead to POGU (40). In addition, the excessive use of online games can also positively predict depressive symptoms and mental health 1 year later (41, 42). However, few studies have explored the relationship between parenting style, depressive symptoms, and POGU at the same time, and only a few western scholars have discussed the impact of early family factors on subsequent externalization of adolescent internalization. For instance, positive family environments, history of family depression, and family substance abuse have a cascade effect on subsequent substance abuse through individual internalization (43). Therefore, we speculate that parenting style and POGU can negatively predict each other through depressive symptoms.

## The Present Study

In summary, based on developmental systems theory and developmental contextualism, the current study constructed an autoregressive cross-lagged model to investigate the direct and indirect relationships between parenting style, depressive symptoms, and POGU in a 1-year longitudinal study conducted among Chinese adolescents. In order to determine the nature and direction of the associations more precisely, we examined four specific competing models. Model 1 was autoregressive and included stability paths between repeated measures. Model 2 was unidirectional, adding the paths from parenting style at an earlier time point to depressive symptoms at a later time point and the paths from depressive symptoms at an earlier time point to POGU at a later time point. Model 3 was bidirectional, adding the paths from depressive symptoms at an earlier time point to parenting style at a later time point and the paths from POGU at an earlier time point to depressive symptoms at a later time point, based on Model 2. Model 4 was a developmental cascades model, including the diagonal paths between constructs at adjacent time points. This model assumed within-variable stability and potential relations among variables, both concurrently and longitudinally, between adjacent time points. We got two hypotheses: (1) parenting style, depressive symptoms, and POGU can significantly predict each other; and (2) depressive symptoms serve as a mediator in the path from parenting style to POGU, and the indirect effect of POGU on parenting style *via* depressive symptoms is also significant.

## Materials and Methods

### Participants and Procedure

By using a simple random cluster sampling approach, 1,100 students from Grade 7 and Grade 8 in Hubei, China were selected as the initial research participants, and the survey was conducted every 6 months. Three measurements were carried out in October 2018 (T1), April 2019 (T2), and October 2019 (T3), respectively. Moreover, the eligible participants were selected based on the following criteria: (1) participants who were adolescents, (2) adolescents who received consent from their guardians to participate, and (3) adolescents who agreed to participate. Omitting participants who dropped out due to various reasons, such as transfer, moving, and going on leave, the remaining number of participants for the three measurements were 1,041 (M_age_ = 12.90 ± 1.324 years; 482 boys and 559 girls), 951(M_age_ = 13.39 ± 0.788 years; 431 boys and 520 girls), and 903 (M_age_ = 14.02 ± 2.504 years; 410 boys and 493 girls), respectively. Results from attrition analyses indicated no significant differences among any of the study variables between children who participated in all waves and those who did not.

The present study was approved by the Research Ethics Committee of the College of Education and Sports Sciences, Yangtze University. Prior to the formal investigation, participants and their parents or legal guardians were provided with written consent forms, which informed them that personal information would be kept confidential and their responses would be used only for research purposes. The questionnaires were distributed by trained data collectors in the classrooms of the participants. Prior to each assessment, the data collectors informed the participants that they could hand over the incomplete questionnaires at any time if they felt uncomfortable.

### Measurement

#### Parenting Style

We used the Chinese version of the simple Egma Minnen av Bardndosnauppforstran Questionnaire (S-EMBU-C) compiled by Perris et al. (44) and translated and revised by Jiang et al. (45) to assess the parenting style of teenagers. The questionnaire consisted of 42 items (21 items in father's version and 21 items in mother's version) rated on a four-point scale (from 1 = never done to 4 = always). Both father's version and mother's version were composed of three dimensions: rejection, emotional warmth, and overprotection, and the three dimensions contained 6, 7, and 8 items, respectively (e.g., “Father/mother often treat me in a way that embarrasses me”). A higher mean score of a particular dimension indicated that the parents adopted the corresponding parenting style more frequently. The questionnaire was widely used in the study of Chinese adolescents and had good reliability and validity (46). In the current study, Cronbach's alpha values for this questionnaire at T1, T2, and T3 were 0.857, 0.840, and 0.862, respectively.

#### Depressive Symptoms

We used the Center for Epidemiologic Studies Depression Scale (CES-D), compiled by Radloff (47) and revised by Chen et al. (48), to assess adolescent frequency of depressive symptoms over the last week (e.g., “I am bothered by some trifles”). The scale consisted of 20 items rated on a four-point score (from 1 = occasionally or not done to 4 = most of the time or duration). A higher mean score indicated a more severe depressive symptom. This measure demonstrated good reliability and validity among Chinese adolescents (49). In this research, Cronbach's alpha values of this scale at T1, T2, and T3 were 0.853, 0.877, and 0.886, respectively.

#### Problematic Online Game Use

We used the Chinese version of problematic online game use questionnaire compiled by Gentile et al. (50), and translated and revised by Yu et al. (51), to assess the frequency of POGU in participants over the past 6 months (e.g., “Have you ever tried to play lesser Internet games, but without success?”). The questionnaire consists of 11 items rated on a three-point score (from 0 = never to 2 = frequently). Scores were recorded as: 0 = “never,” 0.5 = “sometimes,” and 1 = “frequently.” This scoring method allowed taking into account participants who occasionally experienced symptoms, thus increasing accuracy (51). A higher mean score indicated a stronger tendency of POGU, and the cut-off score for identifying adolescents with POGU was 5 or more (2). This measure demonstrated good reliability and validity among Chinese adolescents (52, 53). In this study, Cronbach's alpha values of this scale at T1, T2, and T3 were 0.808, 0.809, and 0.853, respectively.

### Statistical Analysis

First, a series of mixed repeated measures MANOVAs was conducted to examine overall effects of Gender, Time, and their interactions with each variable by SPSS version 24.0 (54). Second, Pearson correlations were calculated among parenting style, depressive symptoms, and POGU based on three measurements. Third, the cascade effects among the main variables were tested using autoregressive cross-lagged panel models (CLPM) by MPLUS version 7.4.

Research shows that children and adolescents are less able to classify parental behavior and parenting style compared to adults, and the structures of parenting style are not yet fully clear (55). Separate analysis of each dimension will lead to a correlation that is too low or not significant. According to the previous research on S-EMBU-C (56, 57), in order to facilitate the analysis and discussion, the three dimensions of parenting style were rotated into two high-order factors: parental care and parental control. The results showed that 33.33% of the variance of the original variable could be explained. Parental control could explain 19.065% of the variance and it mainly had relatively high factor loadings on mother's overprotection, mother's rejection, father's overprotection, and father's rejection. Parental care could explain 14.265% of the variance and it mainly had relatively high factor loadings on mother's emotional warmth and father's emotional warmth. This is consistent with previous studies (58). In the following study, we will examine the relationship between these two dimensions of parenting style, depressive symptoms, and POGU.

To determine model fit, we used the comparative fit index (CFI), the Tucker–Lewis index (TLI), the root mean square error of approximation (RMSEA), the standardized root mean square residual (SRMR), and the Akaike information criterion (AIC). The CFI and TFI at 0.90 or above, and the RMSEA and SRMR at 0.08 or lower, indicated that the model was acceptable. A better fitting model was indicated by a lower AIC value (59). Since the χ2 test of significance is sensitive to large samples it was reported but not used as a measure of absolute model fit (60). An MLR estimator was used to account for potential issues of non-normal data. Missing data were handled using full information maximum likelihood estimation.

## Results

### Test for Common Method Bias

The Harman single factor test was used to test for common method bias (60). The results showed that there were 20 factors with characteristic root values >1 at T1, and the variation value explained by the first factor was 15.512%. There were 19 factors with characteristic root values >1 at T2, and the variation value explained by the first factor was 16.066%. There were 17 factors with characteristic root values >1 at T3, and the variation value explained by the first factor was 16.979%. The variation values were all less than the critical criterion of 40%, indicating that the common method of the study was not effective.

### Descriptive Statistics and Correlations

Means and standard deviations for all the variables at each time point are presented in Table 1. Results of mixed repeated measures MANOVAs indicated that boys scored higher on parental care: *F*_(12,894)_ = 10.532, *p* < 0.01, parental control: *F*_(12,894)_ = 6.057, *p* < 0.05, and POGU: *F*_(12,894)_ = 136.543, *p* < 0.001, and lower on depressive symptoms: *F*_(12,894)_ = 11.250, *p* < 0.01. Depressive symptoms at T1 were significantly lower than at T2, which was significantly lower than at T3: *F*_(22,893)_ = 22.808, *p* < 0.001. The interaction between gender and time of depressive symptoms was significant: *F*_(22,893)_ = 3.580, *p* < 0.05. POGU at T1 was significantly higher than at T2, which was significantly lower than at T3: *F*_(22,893)_ = 4.914, *p* < 0.01. The interaction between gender and time of POGU was significant: *F*_(22,893)_ = 37.505, *p* < 0.001. Other main effects and interactions were not significant.

**Table 1:** Descriptive statistics for main study variables.

	**Boys**		**Girls**	
	***M***	***SD***	***M***	***SD***
**Parental care**
T1	2.815	0.622	2.731	0.696
T2	2.825	0.621	2.718	0.677
T3	2.738	0.635	2.688	0.678
**Parental control**
T1	1.880	0.353	1.834	0.370
T2	1.866	0.346	1.776	0.373
T3	1.823	0.372	1.802	0.366
**Depressive symptoms**
T1	1.739	0.426	1.802	0.475
T2	1.763	0.457	1.887	0.538
T3	1.921	0.495	1.922	0.552
**POGU**
T1	0.552	0.359	0.329	0.297
T2	0.504	0.328	0.294	0.288
T3	0.433	0.340	0.442	0.326

Intercorrelations among all the study variables are presented in Table 2. The results showed that all the variables displayed considerable correlation and stability over a period of 1 year. At each time point, parental care was significantly negatively correlated with depressive symptoms and POGU, parental control was significantly positively correlated with depressive symptoms and POGU, and depressive symptoms were positively correlated with POGU.

**Table 2:** Intercorrelations among study variables.

	**1**	**2**	**3**	**4**	**5**	**6**	**7**	**8**	**9**
**Parental care, Depressive symptoms, and POGU**
T1 Parental care	1								
T1 Depressive symptoms	−0.349^**^	1							
T1 POGU	−0.164^**^	0.297^**^	1						
T2 Parental care	0.065^**^	−0.101^**^	−0.310^**^	1					
T2 Depressive symptoms	−0.205^**^	0.265^**^	0.769^**^	−0.322^**^	1				
T2 POGU	−0.038	0.051	0.286^**^	−0.133^**^	0.299^**^	1			
T3 Parental care	0.042	−0.035	−0.146^**^	0.029^**^^*^	−0.140^**^	−0.034	1		
T3 Depressive symptoms	−0.070^*^	0.046	0.244^**^	0.015	0.251^**^	0.066^*^	−0.286^**^	1	
T3 POGU	−0.158^**^	0.218^**^	0.786^**^	−0.262^**^	0.791^**^	0.232^**^	−0.146^**^	0.228^**^	1
**Parental control, Depressive symptoms, and POGU**
T1 Parental control	1								
T1 Depressive symptoms	0.294^**^	1							
T1 POGU	0.222^**^	0.297^**^	1						
T2 Parental control	0.107^**^	0.139^**^	0.295^**^	1					
T2 Depressive symptoms	0.226^**^	0.265^**^	0.769^**^	0.304^**^	1				
T2 POGU	0.059	0.051	0.286^**^	0.238^**^	0.299^**^	1			
T3 Parental control	0.068^*^	0.069^*^	0.338^**^	0.102^**^	0.348^**^	0.083^*^	1		
T3 Depressive symptoms	0.029	0.046	0.244^**^	0.059	0.251^**^	0.066^*^	0.313^**^	1	
T3 POGU	0.203^**^	0.218^**^	0.786^**^	0.293^**^	0.791^**^	0.232^**^	0.266^**^	0.228^**^	1

* *p < 0.05*,

** *p < 0.01*.

### Test for Cascade Effects

#### Parental Care, Depressive Symptoms, and POGU

**Figure 1:**
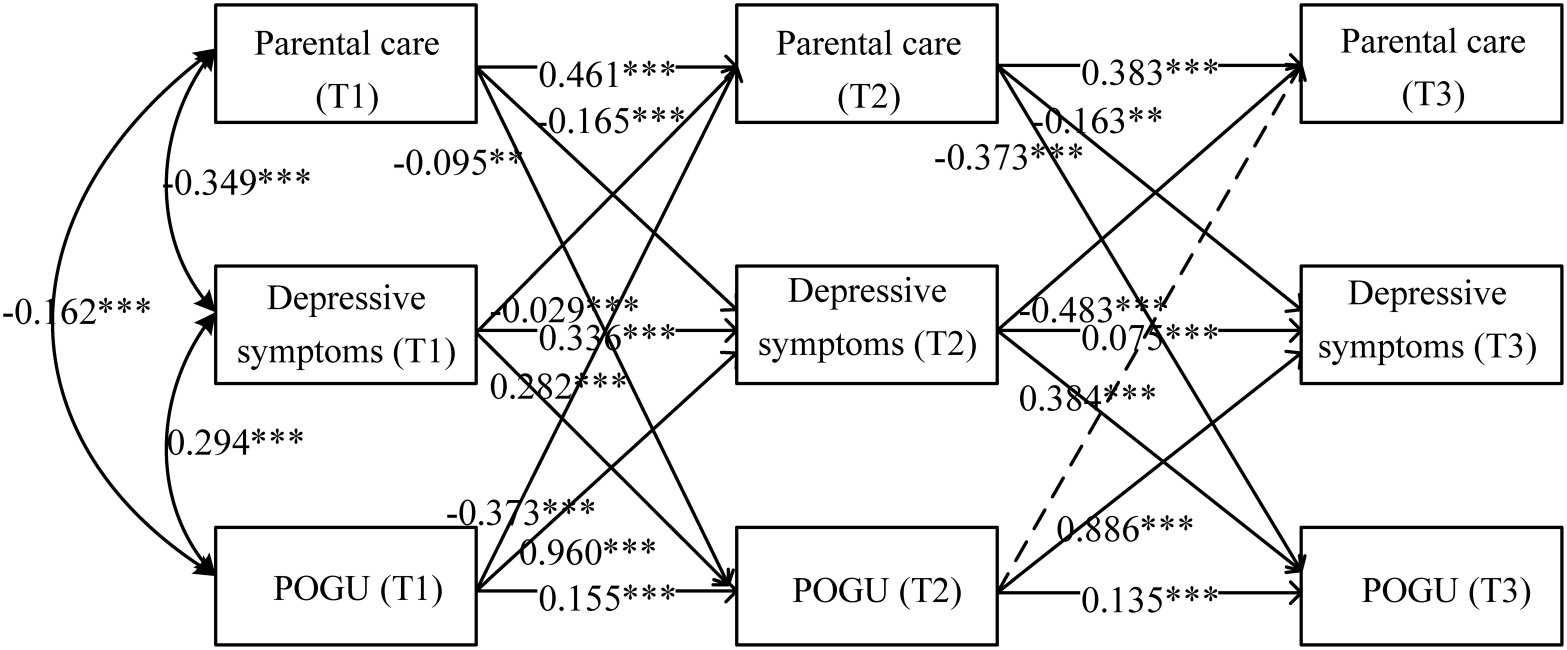
Developmental cascades model of parental care, depressive symptoms, and POGU at T1, T2, and T3. POGU, problematic online game use. Dashed lines represent non-significant paths. The path coefficients are standardized coefficients. **p < 0.01, ***p < 0.001.

Absolute fit indices and model comparison tests are presented in Table 3. The best fitting model was the full model, which is shown in Figure 1. Overall, only POGU at T2 was found insignificant in predicting parental care at T3, meanwhile, all the other paths could be significantly predicted. We then examined the indirect effects of parental care, depressive symptoms, and POGU over 1 year, based on the full model. Overall, the result in Table 4 showed that parental care at T1 could significantly and negatively predict POGU at T3 through depressive symptoms at T2. Moreover, POGU at T1 could predict parental care at T3 through depressive symptoms at T2.

**Table 3:** Model fit indices of parental care, depressive symptoms, and POGU.

	***df***	***c***	***χ^2^***	**CFI**	**TLI**	**RMSEA**	**SRMR**	**AIC**	**Model comparison**	**cd**	**Δ*χ^2^***	**Δ*df***
1. Autoregressive model	18	3.064	374.039	0.951	0.903	0.138	0.071	9816.823				
2. Unidirectional model	14	2.971	148.878	0.982	0.953	0.096	0.123	9545.165	1 vs. 2	3.390	207.165^***^	4
3. Bidirectional model	10	2.867	131.398	0.983	0.940	0.108	0.111	9525.411	2 vs. 3	3.231	20.204^***^	4
4. Full model	6	1.224	11.704	0.999	0.994	0.030	0.004	6606.465	3 vs. 4	5.333	233.20^***^	4

*c weighting constant for computing chi-square under MLR, cd weighting constant for comparing difference in chi-square values using MLR*,

*** *p < 0.01*.

**Table 4:** Indirect effects of parental care, depressive symptoms, and POGU.

	***Effect***	***SE***	***95% CI***
Parental care (T1) to depressive symptoms (T3)	−0.172^**^	0.034	(−0.239, −0.105)
Parental care (T1) to POGU (T3)	−0.248^**^	0.041	(−0.328, −0.168)
Depressive symptoms (T1) to parental care (T3)	−0.173^***^	0.012	(−0.197, −0.149)
Depressive symptoms (T1) to POGU (T3)	0.178^***^	0.037	(0.105, 0.251)
POGU (T1) to parental care (T3)	−0.599^***^	0.006	(−0.611, −0.587)
POGU (T1) to depressive symptoms (T3)	0.266^**^	0.033	(0.201, 0.331)

** *p < 0.01*,

*** *p < 0.001*.

#### Parental Control, Depressive Symptoms, and POGU

**Figure 2:**
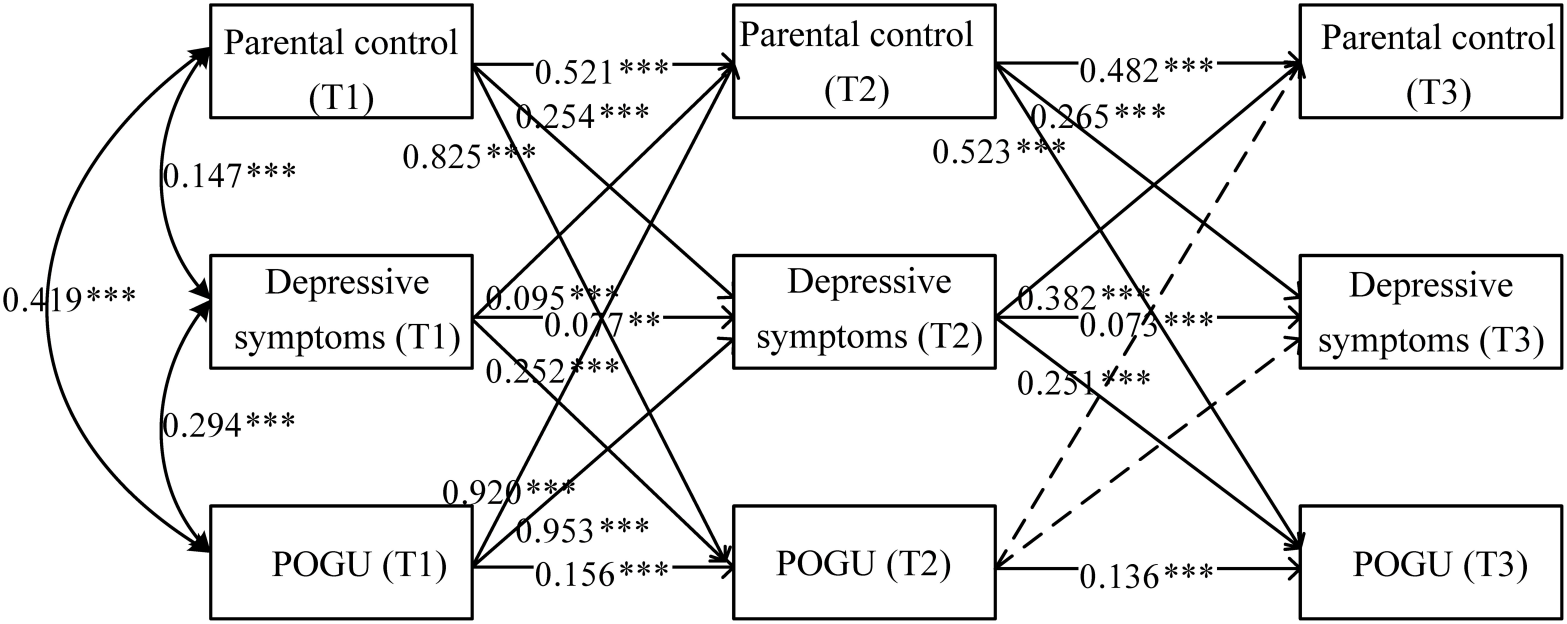
Developmental cascades model of parental control, depressive symptoms, and POGU at T1, T2, and T3. POGU, problematic online game use. Dashed lines represent non-significant paths. The path coefficients are standardized coefficients. **p < 0.01, ***p < 0.001.

Absolute fit indices and model comparison tests are presented in Table 5. The best fitting model was the full model, which is shown in Figure 2. Overall, only POGU at T2 was found insignificant in predicting parental control and depressive symptoms at T3, meanwhile, all the other paths could be significantly predicted. We then examined the indirect effects of parental control, depressive symptoms, and POGU over 1 year, based on the full model. Overall, the result in Table 6 showed that parental control at T1 could significantly and positively predict POGU at T3 through depressive symptoms at T2. Moreover, POGU at T1 could predict parental control at T3 through depressive symptoms at T2.

**Table 5:** Model fit indices of parental control, depressive symptoms, and POGU.

	***df***	***c***	***χ^2^***	**CFI**	**TLI**	**RMSEA**	**SRMR**	**AIC**	**Model comparison**	**cd**	**Δ*χ^2^***	**Δ*df***
1. Autoregressive model	18	1.211	510.162	0.916	0.831	0.162	0.248	4707.630				
2. Unidirectional model	14	1.238	106.216	0.984	0.959	0.080	0.069	4188.230	1 vs. 2	1.117	435.631^***^	4
3. Bidirectional model	10	1.237	90.915	0.986	0.950	0.088	0.046	4176.778	2 vs. 3	1.241	16.040^**^	4
4. Full model	6	1.266	3.984	1.000	1.000	0.001	0.005	3832.598	3 vs. 4	1.194	89.024_^***^_	4

*c weighting constant for computing chi-square under MLR, cd weighting constant for comparing difference in chi-square values using MLR*,

** *p < 0.01*,

*** *p < 0.001*.

**Table 6:** Indirect effects of parental control, depressive symptoms, and POGU.

	***Effect***	***SE***	***95% CI***
Parental control (T1) to depressive symptoms (T3)	0.083^***^	0.033	(0.018, 0.148)
Parental control (T1) to POGU (T3)	0.448^*^	0.012	(0.424, 0.472)
Depressive symptoms (T1) to parental control (T3)	0.075^**^	0.008	(0.059, 0.091)
Depressive symptoms (T1) to POGU (T3)	0.103^***^	0.010	(0.083, 0.123)
POGU (T1) to parental control (T3)	0.807^***^	0.032	(0.744, 0.870)
POGU (T1) to depressive symptoms (T3)	0.314^*^	0.028	(0.259, 0.369)

* *p < 0.05*,

** *p < 0.01*,

*** *p < 0.001*.

## Discussion

Based on developmental system theory and developmental contextualism, this study examines the dynamic interaction model among parenting style, depressive symptoms, and POGU. The results support the developmental cascades model, such that difficulties in a particular domain (e.g., parenting style) have a spreading effect, propagating into other domains (e.g., depressive symptoms and POGU). These observations expand the understanding of the complex relations involving parenting style, depressive symptoms, and POGU among teenagers in China and provide reference suggestions for the prevention and intervention of depression and POGU in adolescents.

## The Characteristics of Parenting Style, Depressive Symptoms, and Adolescent POGU

The results of the preliminary analysis revealed significant gender differences in parental care (e.g., emotional warmth), parental control (e.g., rejection, overprotection), depressive symptoms, and problematic online game use. Girls reported higher depressive symptoms and lower POGU, which were consistent with previous studies (61–63). However, boys reported higher parental care and parental control, which was inconsistent with most studies (56, 64). Although some studies have found that there is no significant gender difference in parental support among adolescents (56), most studies indicate that parents consider adolescent girls to be more vulnerable, and hence give them more care, while boys are considered to be more rebellious and hence shown more restraint (64). One possible explanation is that in Chinese culture, there is a stereotypical mindset that boys become the providers of the family in adulthood (65), and hence, parents pay more attention to boys. Another possible explanation is that self-report parenting style is adopted, which from the perspective of teenagers, makes it difficult to correctly judge parental attitude toward themselves.

## Longitudinal Relationship Between Parenting Style, Depressive Symptoms, and Adolescent POGU

Results from correlations and autoregressive models demonstrated stability in parenting style, depressive symptoms, and POGU over a period of 1 year. After controlling this stability and the intercorrelations among variables at similar time points, cross-lagged associations were observed. Consistent with previous studies, it was observed that parenting style and adolescent depressive symptoms could predict each other over time in both the models. It was also observed that parental care could significantly negatively predict depressive symptoms (66). The cognitive model (67) indicated that positive family factors (e.g., parental care) could promote adolescents to form positive cognition, thus reducing the level of depressive symptoms. Parental control was found to significantly positively predict depressive symptoms, which supports the view of Wood et al. (68). Diathesis stress model holds that depressive symptoms are affected by individual susceptibility and external effects, and parental control can promote the level of individual susceptibility, thus enhancing the effect of stress on depressive symptoms (69). Adolescents living in a family environment of rejection and overprotection over a long period of time experience inferiority and lose self-control, and then produce negative emotions such as depressive symptoms. On the other hand, depressive symptoms can significantly predict parenting style, which is consistent with the social coercion theory (70). Parents and children are easily influenced by each other and become part of a cycle of mutual influence.

The effect of parenting style's prediction of adolescents with POGU was stable, and the effect of POGU on parenting style was also significant but not stable over time in both the models. Parenting style is an important factor affecting adolescents with POGU (9). Negative parenting style may hinder the communication of adolescents with their family and make them lose support from their family, which becomes an alternative reason for them to indulge in Internet games (71). At the same time, the effect of POGU on subsequent parenting style was not stable over time; POGU observed in the first semester of seventh grade predicted parenting style only in the second semester of seventh grade. One possible reason is that teenagers in the first semester of seventh grade would have just entered junior high school, and their parents will pay more attention to them out of concern or worry. Therefore, the parents are more likely to be affected by adolescent problem behaviors.

The effect of depressive symptoms' prediction of adolescents with POGU is stable, and the effect of POGU on depressive symptoms is also significant but not stable over time in both the models. People rely on the Internet to relieve their daily stress, but excessive use of the Internet decreases social skills, further aggravating depressive symptoms (72). Such depressed individuals lose their sense of satisfaction and control in real life, and hide themselves in the virtual world to relieve their depressive symptoms (73). The model of compensatory Internet use shows that depressive symptoms stimulate online motivation and alleviate the negative emotions caused by depressive symptoms (74). Only the prediction of POGU in the second semester of seventh grade to depressive symptoms in the first semester of eighth grade was not significant in the model including parental control. One possible reason is that compared with the second semester of the seventh grade, teenagers who have just entered junior high school would have not yet adapted to the new class and environment, and would have not formed a stronger connection in reality and get less social support, which is more likely to aggravate the level of depressive symptoms. In general, H1 is verified.

## Cascade Effects

In the developmental cascades model, we also explore the indirect relationship among variables. The study found that parental care, parental control, and adolescents with POGU can indirectly and respectively predict each other *via* depressive symptoms, and hence H2 is verified. This result confirms cascade effects which predict that effects of child behaviors in one domain of development can affect other domains over time, via a combination of direct/indirect and unidirectional/bidirectional pathways. From the perspective of cultural psychology, social interactions and behaviors of children are evaluated in the context of cultural norms and values (15). The cultural values endorsed by a family have a role in shaping the family's reactions toward the characteristics and behaviors of their children. Consistent with Patterson's social coercion theory (75), adolescent problem behavior can also affect parenting style.

Parental care and POGU can predict each other *via* depressive symptoms. If parents often provide care, coordination, support, and acquiescence to the needs of their teenage children, the teenagers will, in turn, feel warmth, comfort, care, support, understanding, and love from their parents, thus strengthening the psychological connection and satisfying their psychological needs, and hence the possibility of depressive symptoms will be lower (76, 77). Positive and optimistic teenagers tend to be more willing to interact in real life, pay more attention to controlling their online time, and are less likely to indulge in online games (78). On the other hand, if teenagers independently control the time that they spend on playing online games, they will allot more time studying and getting along with their parents and friends. As a result, the rising academic performance and having more friends will reduce the degree of depressive symptoms, and such children will also be closer to and more loved by their parents in the family, thus receiving attention and parental care.

Parental control and POGU can predict each other *via* depressive symptoms. Parents over-protect, reject, or alienate their children and control them to think, feel, or do things according to their wishes, which decreases self-awareness and increases the sense of helplessness among adolescents, thereby making them more depressed (79, 80). Teenagers with depressive symptoms are more likely to indulge in POGU if they are unable to satisfy their psychological needs in real life (81). In turn, teenagers addicted to POGU often display low social interaction, thus increasing their depressive symptoms. In Chinese culture, teenagers feel ashamed of depressive symptoms, which is often accompanied by problem behavior and interpersonal alienation rather than venting out negative emotions in a proper manner. Moreover, the response of Chinese parents to their children's problem behavior is often more stringent and over-protective (82).

## Limitations and Future Directions

There are several limitations to the current study. First, although this study conducted three longitudinal waves of assessment and found the longitudinal relations between parenting style, depressive symptoms, and POGU, the duration of the longitudinal assessment was still relatively short, and the waves of longitudinal assessment remained to be increased. Second, strictly speaking, the study explored problematic online game use and depression symptoms at the non-clinical level. Future research should use more standardized measurement tools to reveal the relationship at the clinical level. Third, self-reports of parenting style from teenagers were biased. Future research should incorporate data from multiple informants using different methods. Fourth, the sample was limited to adolescents drawn from two junior middle schools in China. Thus, caution is warranted in generalizing the findings to other cultures. Fifth, we did not take into account divorced families, family income, and other variables that may have an effect on depressive symptoms and problematic online game use as covariables. Hence, future research could include covariates to try to get a more accurate conclusion. Finally, the development cascades model did not separate the between-person effects and within-person effects. Therefore, the conclusions of the study cannot be easily generalized at the individual level, and future studies need to further explore the relationship between variables from the person-centered perspective.

## Conclusions

The current results are an extension of previous studies and indicate that the reciprocal effect of parenting style, depressive symptoms, and POGU was significant. Moreover, depressive symptoms serve as a mediator in the path from parenting style to POGU, and the indirect effect of POGU on parenting style via depressive symptoms was significant. These findings enhance the understanding of the complex relations among parenting style, depressive symptoms, and POGU among teenagers in China, and provide reference suggestions for the prevention and intervention of depression and POGU in adolescents.

## Data Availability Statement

The raw data supporting the conclusions of this article will be made available by the authors, without undue reservation.

## Ethics Statement

The studies involving human participants were reviewed and approved by The Research Ethics Committee of College of Education and Sports Sciences, Yangtze University. Written informed consent to participate in this study was provided by the participants' legal guardian/next of kin. Written informed consent was obtained from the individual(s), and minor(s)' legal guardian/next of kin, for the publication of any potentially identifiable images or data included in this article.

## Author Contributions

XG, ML, and CY designed the study. XG, ML, and XJ collected the data. XG, HL, and CZ analyzed the data results and drafted the manuscript. XG, HL, ML, CY, XJ, CZ, and YL revised the manuscript.

## Funding

The present study was funded by the Youth project of the Ministry of Education in 2020 in the 13th Five-Year Plan of the National Education Science: The heterogeneous developmental trajectory of Internet gaming disorder in adolescents: the combined action of parenting environment and genetic polymorphism (EBA200391).

## Conflict of Interest

The authors declare that the research was conducted in the absence of any commercial or financial relationships that could be construed as a potential conflict of interest.

## Publisher's Note

All claims expressed in this article are solely those of the authors and do not necessarily represent those of their affiliated organizations, or those of the publisher, the editors and the reviewers. Any product that may be evaluated in this article, or claim that may be made by its manufacturer, is not guaranteed or endorsed by the publisher.
